# Misindentification of *Mycobacterium kumamotonense* as *M. tuberculosis*

**DOI:** 10.3201/eid1607.091913

**Published:** 2010-07

**Authors:** Almudena Rodríguez-Aranda, María S. Jiménez, Jesús Yubero, Fernando Chaves, Rafael Rubio-García, Elia Palenque, María J. García, M. Carmen Menéndez

**Affiliations:** Author affiliations: Hospital Universitario Doce de Octubre, Madrid, Spain (A. Rodríguez-Aranda, F. Chaves, R. Rubio Garcia, E. Palenque);; Instituto de Salud Carlos III, Madrid (M.S. Jiménez);; Universidad Autónoma de Madrid, Madrid (J. Yubero, M.J. García, M.C. Menéndez)

**Keywords:** Mycobacterium kumamotonense, M. tuberculosis, tuberculosis and other mycobacteria, misidentification, bacteria, diagnostic tests, letter

**To the Editor:** Because of slow growth of mycobacteria, use of rapid tests to identify them is strongly recommended; rapid tests are widely used as an advanced diagnostic tool in clinical laboratories (*1**,**2*). These tests are particularly useful for diagnosing extrapulmonary mycobacterioses and identifying unusual mycobacteria as etiologic agents (*3*). Commercial probes are frequently used for rapid and specific identification of mycobacteria, especially *Mycobacterium tuberculosis* complex. However, cross-reactivity of DNA probes between mycobacterial species could result in incorrect diagnosis and treatment of patients (*4**,**5*). Misidentification could be a problem if a newly described species, such as *M*. *kumamotonense* (*6*), were an etiologic agent of a disease.

In July 2006, we obtained a fine-needle, puncture aspiration biopsy specimen from a cervical lymph node of a 30-year-old man at Doce de Octubre Hospital (Madrid, Spain). The patient was a recent immigrant from Paraguay and was HIV positive (C2 stage of infection). A biopsy specimen from a cervical lymph node showed necrotizing granulomatous lymphadenopathy. A computed tomographic scan showed cervico-thoraco-abdominal, multiple cervical, supraclavicular, axillar, paratracheal, and mediastinal lymphadenopathies. The patient had a CD4 cell count of 219 cells/mm^3^ and an HIV viral load of 197,181 copies/mL.

The aspiration sample was positive for acid-fast bacilli by fluorescent staining. The clinical isolate (designated 1369) obtained from the aspirate sample was grown in liquid media (MGIT Diagnostic Kit; Becton Dickinson Diagnostics, Sparks, MD, USA) and identified as *M. tuberculosis* complex by using the AccuProbe System (bioMérieux, Marcy l’Etoile, France).

A diagnosis of lymphoid tuberculosis was made, and the patient was treated with isoniazid, rifampin, ethambutol, and pyrazinamide. After 1 month, rifampin was withdrawn because of a cutaneous exanthem. Three months later, the clinical status of the patient had improved, fever had disappeared, and sizes of cervical and axillary lymph nodes had decreased. Treatment with tenofovir, emtricitabine, and lopinavir/ritonavir was started. Two weeks later, an immune reconstitution syndrome and adenopathies developed, but these resolved in 1 month.

Five months after treatment was started, susceptibility testing in a reference laboratory showed that isolate 1369 was *M*. *kumamotonense*. The isolate showed 100% identity with the 16S rRNA gene sequence of *M*. *kumamotonense* (GenBank accession no. AB239925). Results of PCR restriction analysis of heat shock protein 65 gene (*7*) (http://app.chuv.ch/prasite/index.html) were consistent with those for *M*. *kumamotonense*. The isolate was susceptible to ethambutol, rifampin, cycloserine, and ethionamide and resistant to isoniazid, streptomycin, pyrazinamide, and kanamycin.

Because of the improvement in the clinical status of the patient, treatment continued without modification for 18 months. At this time, his CD4 cell count was 488 cells/mm^3^ and his HIV viral load was <50 copies/mL. In July 2009, the patient was asymptomatic and had a CD4 cell count of 631 cells/mm^3^ and an HIV viral load <50 copies/mL.

To confirm misidentification of *M*. *kumamotonense* as a member of the *M. tuberculosis* complex, other commercial probes were tested. Isolate 1369 was also misidentified as *M. tuberculosis* complex by Inno-LIPA v2 (Innogenetics, Ghent, Belgium). The isolate was identified as *Mycobacterium* sp. by Geno-Type (Hain Lifescience, Nehren, Germany). The 3 commercial probes we used had different genome region specificities, all in the mycobacterial ribosomal operon. The AccuProbe System was specific for 16S rDNA, Inno-LIPA v2 was specific for internal transcribed spacer 1, and Geno-Type was specific for 23S rDNA. Only Geno-Type did not show cross-reactivity between *M. tuberculosis* complex and *M*. *kumamotonense*. The clinical isolate was identified as *M*. *kumamotonense*, a new, slow-growing mycobacterium that was first isolated from an immunocompetent patient in Japan (*6*). We showed that this species caused extrapulmonary disease in an HIV-positive patient.

Misidentification of *M*. *kumamotonense* as *M. tuberculosis* complex by commercial DNA probes has serious clinical implications. Once a patient is given a diagnosis of tuberculosis, he or she will be treated with specific drugs for a long period and be prone to adverse side effects. Furthermore, *M*. *kumamotonense* is resistant to many drugs used during typical treatment. After a diagnosis of tuberculosis, patient contacts need to be investigated to identify new cases. Emerging mycobacterial pathogens, such as *M*. *kumamotonense*, may also cause pulmonary and extrapulmonary infections that are also caused by other members of this genus and could be misidentified as *M*. *tuberculosis*.
